# Mesenchymal stem cells decrease blood–brain barrier permeability in rats with severe acute pancreatitis

**DOI:** 10.1186/s11658-019-0167-8

**Published:** 2019-06-17

**Authors:** Ronggui Lin, Ming Li, Meiqin Luo, Tianhong Teng, Yu Pan, Heguang Huang

**Affiliations:** 1 Department of General surgery, Fujian Medical University Union Hospital, 29 Xinquan Road, Fuzhou, Fujian 350001 People’s Republic of China; 2 Department of Histology and Embryology, Hunan University of Medicine, Huaihua, Hunan China; 3 Department of Orthopedics, Fujian Medical University Union Hospital, Fuzhou, Fujian China

**Keywords:** Severe acute pancreatitis, Blood–brain barrier, Mesenchymal stem cell, Inflammatory response, Endothelial cell

## Abstract

**Background:**

Impairment of the blood–brain barrier (BBB) could result in secondary cerebral edema and life-threatening pancreatic encephalopathy in patients with severe acute pancreatitis (SAP). Mesenchymal stem cells (MSCs) have been widely adopted in clinical research because of their pleiotropic functions. The aim of this study was to investigate the impact of MSCs on BBB permeability in SAP and the potential mechanisms driving these effects.

**Methods:**

Sprague-Dawley rats were randomly assigned to the control, SAP and SAP+MSCs groups. Pancreatic impairment was assessed. The serum levels of amylase, TNF-α and IL-10, expression levels of claudin-5, Bax, Bcl-2 and MMP-9, and the BBB permeability were measured. Endothelial cell apoptosis was evaluated.

**Results:**

SAP rats showed BBB impairment with increased permeability and secondary cerebral edema, which was confirmed using the Evans blue assay and the calculation of the brain dry/wet ratio. Treatment with MSCs decreased the serum levels of amylase and TNF-α, increased the serum levels of IL-10, attenuated the apoptosis of brain microvascular endothelial cells, upregulated claudin-5 expression and downregulated MMP-9 expression. This treatment attenuated the increased BBB permeability in SAP rats.

**Conclusions:**

MSCs attenuated the impairment of the BBB and decreased its permeability, producing protective effects in SAP rats.

**Electronic supplementary material:**

The online version of this article (10.1186/s11658-019-0167-8) contains supplementary material, which is available to authorized users.

## Background

Pancreatic encephalopathy (PE) is a rare and serious complication of severe acute pancreatitis (SAP) presenting with psychogeny, with a high mortality rate of approximately 67% [1]. The underlying pathogenesis remains to be explored, but the main promising hypothesis suggests that PE development is closely related to a blood–brain barrier (BBB) impairment that causes increased permeability.

The BBB protects the central nervous system from pathogens [2]. It is mainly composed of brain microvascular endothelial cells (BMECs), paracellular junctions, astrocytes, pericytes and the basement membrane [3]. BMECs and paracellular junctions are the structural and functional components of the BBB. Paracellular junctions include tight, adherens and gap junctions [4–6], and claudin-5 is a critical tight junction protein for maintaining the integrity and stability of the barrier [7].

Matrix metalloproteinase-9 (MMP-9) is a member of the zinc-dependent metalloproteinase family, which is involved in the degradation of extracellular matrix components. It has been reported that MMP-9 causes increased degradation of collagen IV in the vascular basement membrane and a subsequent increase in BBB permeability in diabetic mice following a stroke [8]. MMP-9 is often upregulated by various inflammatory cytokines and mediators in an aggravated inflammatory response following brain injury, leading to an increase in BBB permeability. However, it is unclear whether MMP-9 is upregulated in SAP rats with increased BBB permeability.

Mesenchymal stem cells (MSCs) have been widely applied in clinical research as a result of their superior multipotential differentiation, low immunogenicity and paracrine function. A recent study demonstrated that they exert immunomodulatory effects during the treatment of various inflammatory diseases [9]. Another study showed that they promote tissue repair and have anticancer properties [10]. In our previous study, we demonstrated that MSCs protect the endothelial barrier in the small intestine and kidney in SAP rats [11, 12]. However, their similar effects on the BBB in SAP rats and the underlying mechanisms remain unclear. In this study, an SAP rat model was established to study the potential mechanisms of impairment of the BBB and the effects of MSCs on this important barrier.

## Materials and methods

### Animals

Germ-free adult male Sprague-Dawley (SD) rats weighing 200–250 g were obtained from Shanghai SLAC Laboratory Animal Co. Ltd. They were allowed to acclimatize for a week in rooms with a 12-h light–dark cycle at a temperature of 20 ± 2 °C. All animal experimental protocols were approved by the Experimental Animals Committee of Fujian Medical University. All experimental animals received humane care and were treated according to ethical guidelines and standards [13].

The rats (*n* = 30) were randomly divided into 3 groups: control, SAP and SAP+MSCs (*n* = 10 per group). An SAP model was established for the rats in the SAP and SAP+MSCs groups. The rats in the SAP+MSCs group received 1 ml of MSCs (approximately 1 × 10^6^ cells/ml) via an injection into the femoral vein after the establishment of the model. All the animals were euthanized after 12 h for sample collection.

### Isolation, culture and identification of MSCs

MSCs were isolated using the differential adherence method, as described previously [14]. The tibia and femur of a 1-month old SD rat were separated, and the bone marrow cavities were exposed and flushed with Dulbecco’s modified Eagle’s medium (DMEM; HyClone). The collected DMEM was centrifuged at 300×g for 5 min, then resuspended, supplemented with 10% (w/v) fetal bovine serum (FBS; Gibco) and 1% penicillin/streptomycin (HyClone). The cells were inoculated in a 25-cm^2^ culture flask at a concentration of 5 × 10^7^ cells/ml, then incubated at 37 °C with 5% CO_2_. Non-adherent cells were removed by washing the flask with phosphate-buffered saline (PBS; HyClone) three times 24 h later. The medium was changed every 2–3 days until the cells reached a confluency of 80–90%. The MSCs were purified by dissociation, and the third generation was used for further experiments.

MSCs were identified using flow cytometry analysis (FACS, Beckton Dickinson) by detecting the cell surface markers CD29, CD34, CD45 and CD90. The respective phycoerythrin-conjugated primary antibodies were a mouse/rat anti-CD29 antibody (1 μg; eBioscience), mouse anti-CD34 antibody (1 μg; Santa Cruz Biotechnology), rat anti-CD45 antibody (0.25 μg; eBioscience) and mouse/rat anti-CD90 antibody (0.06 μg, eBioscience).

### Establishment of an SAP animal model

Retrograde injection of 5% sodium taurocholate (0.1 ml/100 g body weight, Inalco Spa) into the biliopancreatic duct was used to establish an SAP rat model, as described previously [15, 16]. Before the operation, the rats were allowed to drink water but were fasted for 12 h. Anesthesia was performed via an abdominal cavity injection with 10% chloral hydrate (3 ml/kg body weight, Bio Basic). A 1- to 1.5-cm incision in the midline of the upper abdomen was used for the laparotomy. A 0.45-mm diameter polyethylene catheter was used for the injection, which was performed at a speed of 0.04 ml/min using a microinfusion pump that was removed 10 min later. In the control group, the pancreas and duodenum were maneuvered during the laparotomy without any injection into the biliopancreatic duct. The abdomen was closed with sutures, and after the surgery, the rats were given 4 ml normal saline/100 g body weight every 6 h via subcutaneous injection at multiple sites on the back.

### Histological analysis

Four micron sections of the harvested body of the pancreas were stained with hematoxylin and eosin (H&E), then viewed under a light microscope (Carl Zeiss). The pathological changes were evaluated with a pathological scoring system [17].

### Serum amylase detection and enzyme-linked immunosorbent assay (ELISA)

Serum was obtained from the collected blood samples with centrifugation at 3000 x g at 4 °C for 10 min. It was stored at − 80 °C until further analysis. The serum amylase level was measured with an Olympus AV2700 automated clinical biochemistry analyzer. The serum levels of tumor necrosis factor α (TNF-α) and interleukin-10 (IL-10) were detected with a rat ELISA kit (R&D Systems) in accordance with the manufacturer’s instructions.

### Evans blue assay

We prepared another 3 groups of experimental rats following the same experimental procedures to measure the permeability of the BBB using the Evans blue assay as previously described [18]. Evans blue solution (Sigma) at a concentration of 2% at 5 ml/kg body weight was injected into the femoral vein 1 h before the rats were euthanized. The circulating dye was cleared with a perfusion of cold PBS. The harvested brain tissue was homogenized and incubated in dicarboxamide at 37 °C for 48 h. After centrifugation at 300×g for 5 min, the optical density (OD) of the supernatants was measured at 620 nm absorbance with a SpectraMax M5e Multi-Mode Microplate Reader (Molecular Devices).

### Brain dry/wet ratio calculation

The brain dry/wet ratio was calculated to evaluate the severity of cerebral edema. The collected frontal lobe was weighed before and after drying in an oven at 80 °C for 48 h. The ratio was calculated as dry weight (g)/wet weight (g).

### Quantitative real-time PCR

Total RNA was extracted from the brain tissue with Trizol reagent (Life Technologies) followed by reverse transcription into cDNA with a Transcriptor First Strand cDNA Synthesis Kit (Roche) in accordance with the manufacturer’s instructions. Quantitative real-time PCR was conducted with a StepOnePlus Real-Time PCR System (Applied Biosystems) with Bestar SybrGreen qPCR Mastermix (DBI Bioscience). Glyceraldehyde-3-phosphate dehydrogenase (GAPDH) was used as an internal reference. The relative expression of each gene was calculated with the 2^−ΔΔCT^ method. The primer sequences included rat GAPDH, 5′-GCGAGATCCCGCTAACATCA-3′ and 5′-GGCACCGTTGGATCATAG-3′; claudin-5, 5′-GCACTCTTTGTTACCTTGAC-3′ and 5′-GGCACCGTTGGATCATAG-3′; Bcl-2-associated X (Bax), 5′-CAGACGGCAACTTCAACT-3′ and 5′- CTTCCAGATGGTGAGTGA − 3′; and B-cell lymphoma 2 (Bcl-2), 5′-GCAGAGATGTCCAGTCAG-3′; 5′-ATCCACAGAGCGATGTTG-3′(as indicated in Addtional file 1).

### Western blot analysis

Brain tissue was homogenized on ice, and ice-cold RIPA lysis buffer (Beyotime) containing 1 mM PMSF (Beyotime) was added to each sample. After centrifugation at 14,000×g at 4 °C for 10 min, the supernatants were collected. The protein concentration was measured with a BCA kit (Beyotime). Approximately 50 μg of protein was separated via SDS-PAGE with an appropriate concentration of SDS. The protein was then transferred to nitrocellulose membranes. The membranes were incubated with the following primary antibodies at 4 °C overnight: mouse anti-claudin-5 (1:500, Invitrogen), mouse anti-Bax (1:1000, Cell Signaling Technology), rabbit anti-Bcl-2 (1:1000, Abcam), rabbit anti-MMP-9 (1:1000, Abcam) and mouse anti-β-actin (1:1000, Transgen). Then, the membranes were incubated with the appropriate secondary antibodies at 25 °C for 2 h: goat anti-mouse IgG-HRP and goat anti-rabbit IgG-HRP antibodies (1:5000, Cell Signaling Technology). After detection with a ChemiDocTM MP imaging system (Bio-Rad), the results were analyzed with Image J software version version 1.48.

### Immunohistochemistry staining

Following dewaxing, rehydration and antigen retrieval, the 4-μm sections of the brain tissue were incubated with hydrogen peroxide to block endogenous peroxidase, then blocked with 5% bovine serum albumin (BSA; Sigma) for 1 h. The sections were incubated with anti-claudin-5 antibody (1:50, Invitrogen) at 4 °C overnight and then with secondary antibody (1:100; Abcam) for 30 min at 37 °C. Peroxidase activity was visualized with 3-diaminobenzidine (DAB). Then, the slides were stained with hematoxylin, dehydrated with a gradient alcohol, cleared with xylene, and cover slipped.

### Terminal deoxynucleotidyl transferase-mediated nick end labelling (TUNEL) assay

As previously described [19], the sections of brain tissue were incubated with a TUNEL reaction mixture (TUNEL staining kit; Roche) at 37 °C for 1 h following dewaxing, rehydration and blocking endogenous peroxidase. After staining with DAPI (1:1000; Sigma), the sections were viewed under a fluorescence microscope (Carl Zeiss), and the TUNEL- and DAPI-positive cells were counted.

### Statistical analysis

Data are presented as the means ± SD (standard deviation) and were analyzed with the statistical software SPSS 19.0. Differences between the groups were analyzed using one-way analysis of variance (ANOVA) with multiple comparisons. *p* < 0.05 was considered statistically significant.

## Results

### The culture and identification of rat MSCs

**Fig. 1:**
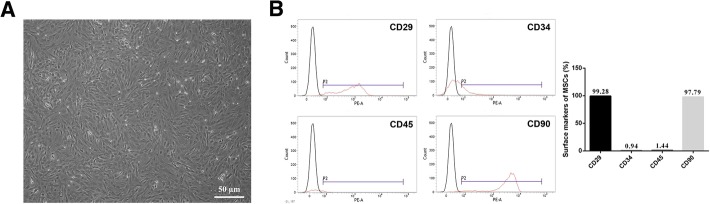
The culture and identification of rat MSCs. **a** Morphology of rat MSCs under a light microscope, scale bar represents 50 μm. **b** Flow cytometry for the detection of the MSC cell surface markers CD29, CD34, CD45 and CD90

The MSCs adhering to the wall of the culture flask 24 h after inoculation were purified via dissociation. The third-generation MSCs were spindle-shaped and adherent (Fig. 1a). After harvesting, the MSCs were identified using flow cytometry for specific cell surface markers, including CD29, CD34, CD45 and CD90. Flow cytometry analysis showed that CD29-, CD34-, CD45- and CD90-positive cells respectively accounted for approximately 99.28, 0.94, 1.44 and 97.79% of the cells, which met the requirements for further experiments (Fig. 1b).

### MSCs alleviated pancreatic impairment and decreased BBB permeability in SAP rats

**Fig. 2:**
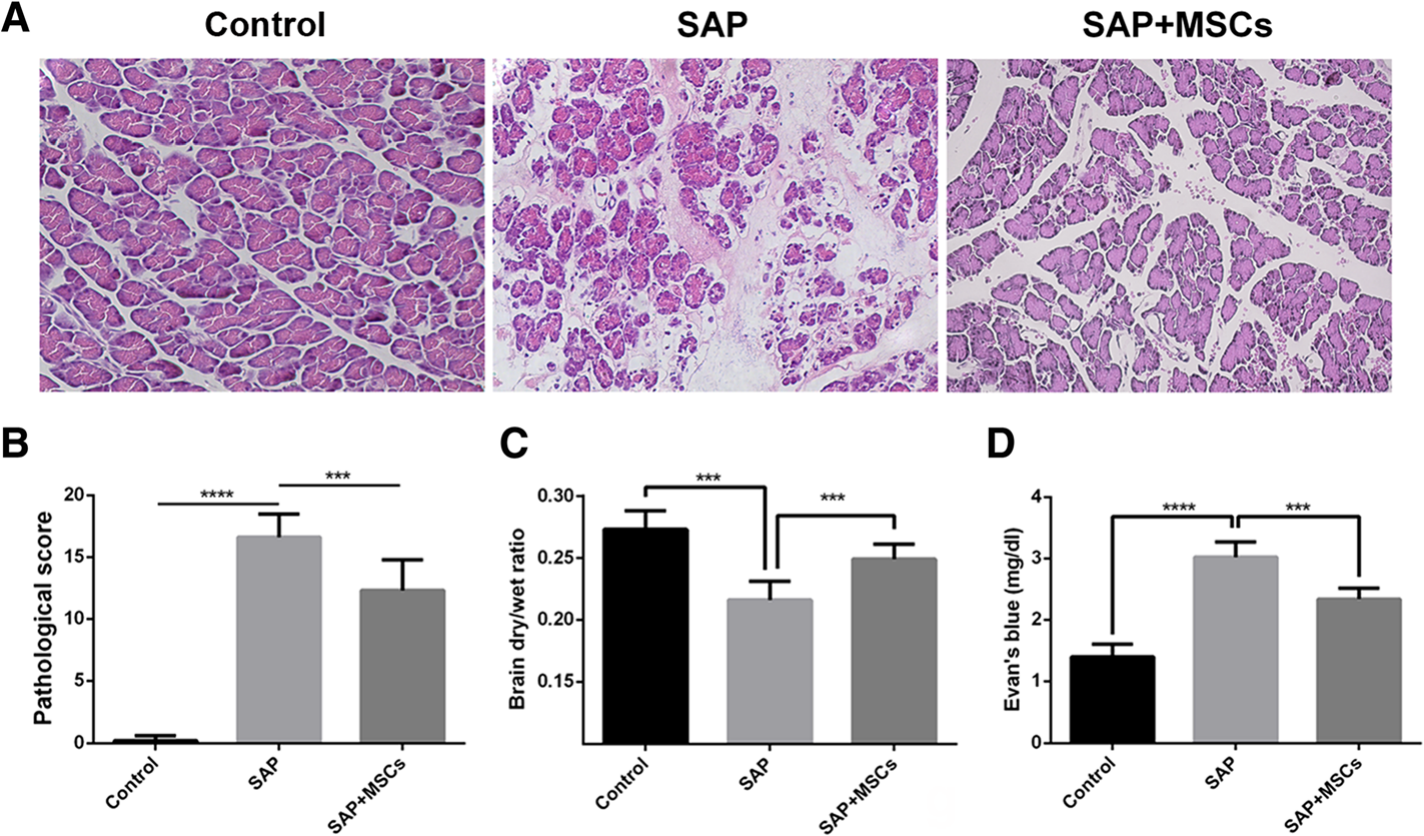
MSCs alleviated pancreatic impairment and decreased BBB permeability in SAP rats. **a** H&E staining of the pancreas (× 200). No obvious pathological changes were observed in the control group, while edema, hemorrhage, necrotic acini and the infiltration of inflammatory cells were observed in the SAP group. The pancreatic impairment in the SAP+MSCs group was milder than that in the SAP group. **b** Pancreatic pathological scores. **c** Brain dry/wet ratio calculation. **d** The results of the Evans Blue assay. ***p < 0.001, ****p < 0.0001)

The typical manifestations of SAP, including ascites and scattered saponification spots on the mesenterium and the greater omentum, were observed when the rats were euthanized. The control group did not show pathological changes in the pancreas based on H&E staining. Pancreatic edema, hemorrhage, necrotic acini and the infiltration of inflammatory cells were observed in the SAP group under a light microscope (Fig. 2a). The pancreatic impairment in the SAP+MSCs group was milder than in the SAP group. In concordance with these changes, the pancreatic pathological scores also showed that the SAP group had a significantly higher score than the control group, and that the score decreased with MSC treatment (*p* < 0.001; Fig. 2b).

To assess the potential impacts of MSCs on the BBB in SAP rats, the Evans blue assay and the brain dry/wet ratio were used to evaluate BBB permeability and secondary cerebral edema. The values of the Evans blue assay for the control, SAP and SAP+MSCs groups were respectively 1.39 ± 0.21, 3.02 ± 0.24 and 2.34 ± 0.18 mg/dl and those for the brain dry/wet ratio were respectively 0.27 ± 0.01, 0.21 ± 0.01 and 0.25 ± 0.01 (Fig. 2c and d). The results showed a higher Evans blue value and a lower brain dry/wet ratio value in the SAP group than in the control group, indicating increased BBB permeability with serious secondary cerebral edema in SAP rats (*p* < 0.001). The changes were partly reversed in SAP rats that received MSC treatment, which showed that MSCs decreased BBB permeability and exhibited protective abilities (p < 0.001).

### MSCs attenuated the severity of systematic inflammation in SAP rats

**Fig. 3:**
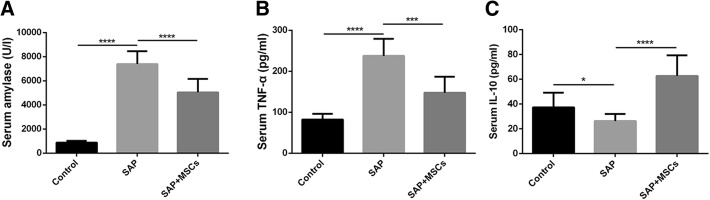
MSCs attenuated the severity of systematic inflammation in SAP rats. **a** Serum amylase levels. **b** Serum TNF-α levels. **c** Serum IL-10 levels. *p < 0.05, ***p < 0.001, ****p < 0.0001

In the control, SAP and SAP+MSCs groups, the levels of serum amylase were respectively 869 ± 154, 7393 ± 1071 and 5042 ± 1119 IU/l; the levels of serum TNF-α were respectively 82.5 ± 13.8, 237.6 ± 41.7 and 147.7 ± 39.2 pg/ml; and the levels of serum IL-10 were respectively 37.4 ± 11.8, 26.3 ± 5.6 and 62.7 ± 16.6 pg/ml (Fig. 3a–c). The serum levels of amylase and TNF-α were higher and the level of IL-10 was lower in the SAP group than those in the control group (*p* < 0.05). In contrast, the serum levels of amylase and TNF-α were lower and the level of IL-10 was higher in the SAP+MSCs group (p < 0.001) than those in the SAP group. These results show that MSCs attenuate pancreatic impairment and decrease the levels of serum amylase and TNF-α while increasing the level of serum IL-10 in SAP rats.

### MSCs decreased the downregulation of claudin-5 in the brains of SAP rats

**Fig. 4:**
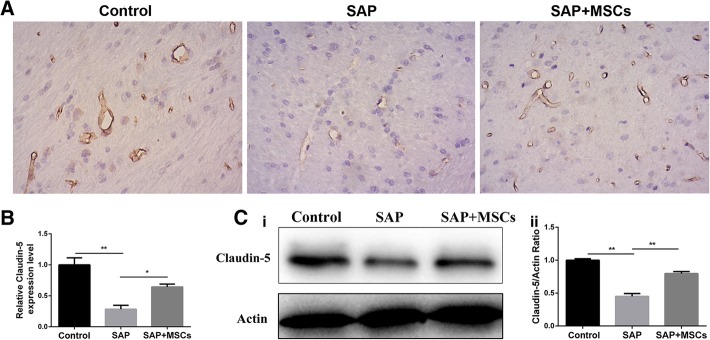
MSCs attenuated the downregulation of claudin-5 in the brains of SAP rats. **a** Immunohistochemistry staining of the brain (× 400). The immunohistochemistry staining shows that there is a higher expression of claudin-5 in BMECs in the control group than in the SAP group. The expression of claudin-5 in the SAP+MSCs group was higher than in the SAP group, although it was lower than in the control group. **b** Results of the quantitative real-time PCR assay of claudin-5 gene expression in the brains of SAP rats. **c** (i) Representative western blotting image of claudin-5 protein expression in the brains of SAP rats. (ii) Statistical analysis of claudin-5 protein expression based on a western blotting assay. *p < 0.05, **p < 0.01

The expression level of the tight junction protein claudin-5 was measured to evaluate for the mechanism leading to the impairment of the BBB. Immunohistochemistry staining showed a higher expression of claudin-5 in BMECs in the control group than in the SAP group. The expression of claudin-5 in the SAP+MSCs group was higher than that in the SAP group, although it was lower than that in the control group (Fig. 4a).

The results of the quantitative real-time PCR and western blotting assays were in accordance with those of the immunohistochemistry staining (Fig. 4b and c). These results revealed that claudin-5 was downregulated in the brains of SAP rats compared to its expression in the controls. This might have contributed to the increased BBB permeability. Treatment with MSCs partly reversed the downregulation of claudin-5, maintaining the stability of the BBB.

### MSCs reduced BMEC apoptosis in the brains of SAP rats

**Fig. 5:**
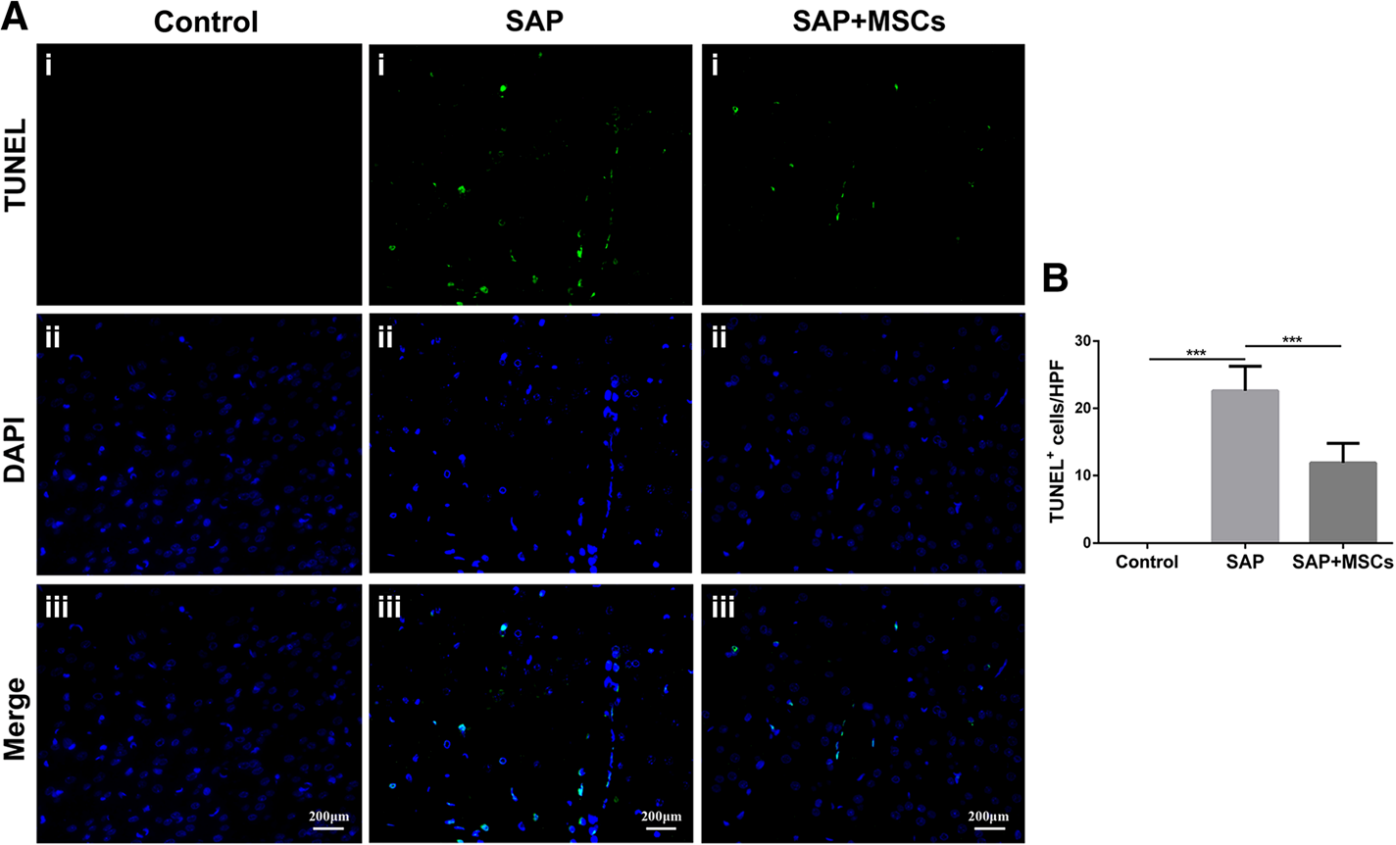
MSCs reduced BMEC apoptosis in the brains of SAP rats**. a** TUNEL staining of the brain, scale bar = 200 μm. No apoptosis was observed in the control group, but numerous apoptotic cells, mainly BMECs, were observed in the SAP group. Fewer apoptotic cells were observed in the SAP+MSCs group than in the SAP group. **b** Statistical analysis of TUNEL-positive cells. ***p < 0.001

TUNEL staining was performed to assess apoptosis in the brains of SAP rats. TUNEL staining revealed no apoptosis in the control group, but numerous apoptotic cells, mainly BMECs, were observed in the SAP group (*p* < 0.001). Fewer apoptotic cells were observed in the SAP+MSCs group than in the SAP group (p < 0.001; Fig. 5). The results show that brain cells become apoptotic in SAP and that treatment with MSCs attenuates apoptosis.

### MSCs upregulated Bcl-2 expression and downregulated Bax expression in the brains of SAP rats

**Fig. 6:**
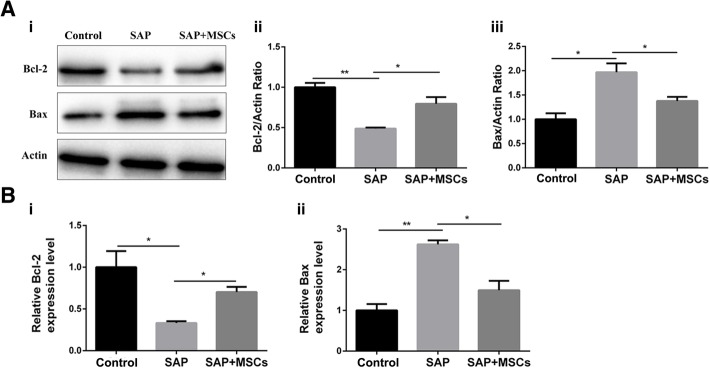
MSCs upregulated Bcl-2 expression and downregulated Bax expression in the brains of SAP rats. **a** (i) Representative western blotting images of Bcl-2 and Bax protein expression in the brains of SAP rats. (ii) Statistical analysis of Bcl-2 protein expression based on a western blotting assay. (iii) Statistical analysis of Bax protein expression based on a western blotting assay. **b** (i) Results of a quantitative PCR assay for Bcl-2 gene expression in the brains of SAP rats. (ii) Results of a quantitative PCR assay for Bax gene expression in the brains of SAP rats. *p < 0.05, **p < 0.01

To explore the potential molecular apoptotic mechanisms, the expression levels of the apoptotic protein Bax and the antiapoptotic protein Bcl-2 were measured. The results of western blotting assays and quantitative real-time PCR revealed that Bax was upregulated and Bcl-2 was downregulated in the SAP group compared with the levels in the control group (*p* < 0.05; Fig. 6a and b).

The results of the TUNEL staining show numerous apoptotic cells, and the upregulation of Bax and downregulation of Bcl-2 are possible stimuli of brain cell apoptosis in the SAP rats. The lower expression level of Bax and higher expression level of Bcl-2 in the SAP+MSCs group than in the SAP group (p < 0.05) indicate that the MSCs partly reversed the modulation of Bax and Bcl-2. In summary, MSCs decreased the upregulation of Bax and the downregulation of Bcl-2 to produce anti-apoptotic effects on brain cells in SAP rats.

### MSCs reduced the upregulation of MMP-9 in the brains of SAP rats

**Fig. 7:**
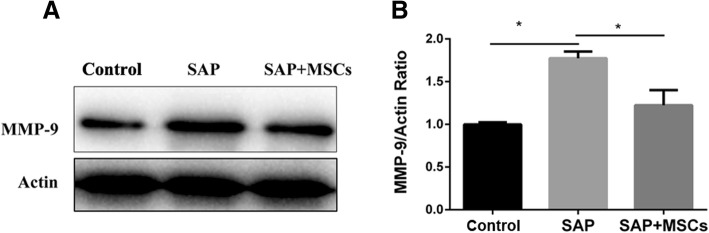
MSCs attenuated the upregulation of MMP-9 in the brains of SAP rats. **a** Representative western blotting image of the MMP-9 protein expression in the brains of SAP rats. **b** Statistical analysis of the MMP-9 protein expression based on a western blotting assay. *p < 0.05

The expression of MMP-9, which has been reported to be a cause of increased BBB permeability, was also measured. A western blotting assay showed that there was a higher expression level of MMP-9 in the SAP group than in the control group (p < 0.05), while the expression level was reduced in the SAP+MSCs group (p < 0.05) (Fig. 7) compared to that in the control group. The results show that MMP-9 was upregulated in the SAP group and that this upregulation was reduced by MSCs.

## Discussion

Severe acute pancreatitis (SAP) is a critical acute abdominal disease characterized by systemic inflammatory response syndrome (SIRS) in the clinical setting [20]. In a cascading reaction, capillary endothelial cells can easily be attacked by a large number of circulating inflammatory cytokines, resulting in capillary leak syndrome (CLS), which is a serious impairment of the endothelial barrier [21, 22].

The blood–brain barrier (BBB) protects the CNS from pathogens. It is an ideal endothelial barrier with very strong barrier properties [23, 24]. Impairment of the BBB refers to CLS in the brain. In SAP, it is associated with increased permeability. The treatment of CLS is a critical component of SAP therapy, and the prevention and treatment of pancreatic encephalopathy (PE) mainly lie in the protection of the BBB. It is essential for reducing complications and decreasing the mortality rate.

Lesions of any component can impair the BBB, meaning a decrease in integrity and function [25] and an increase in permeability. Our study used the Evans Blue assay and the brain dry/wet ratio calculation to confirm the impairment of the BBB in SAP rats with increased permeability and secondary cerebral edema, which is in line with results of a previous study [26].

We also explored the underlying mechanisms of the impairment of the BBB and came to the following conclusions. First, injury or apoptosis of BMECs could cause BBB impairment. The BMECs are directly exposed to the circulating blood flow and could be easily affected by inflammatory cytokines in response to injury or apoptosis during inflammatory situations [27]. In SAP, the remarkably increased levels of serum amylase and inflammatory cytokines, such as TNF-α, might lead to BMEC injury. TUNEL staining revealed numerous apoptotic BMECs in SAP rats, which was in line with previously published reports. In combination with the results of quantitative real-time PCR and western blotting assays, which showed a remarkable increase in Bax expression and a decrease in Bcl-2 expression, we infer that the upregulation of Bax and downregulation of Bcl-2 is a possible mechanism of BMEC apoptosis.

Second, the downregulation of the tight junction protein claudin-5 could contribute to BBB impairment. Claudins are important components that maintain the tight junctions that are responsible for BBB integrity [28, 29]. Claudin-5 is the main transmembrane protein component of tight junctions in BMECs, and it has been reported that claudin-5 is the only molecule whose function is specifically required to maintain the BBB [7]. Previous studies have demonstrated that paracellular tight junctions are damaged [30] and claudin-5 expression was downregulated [31, 32] under inflammatory conditions in vitro and in vivo, resulting in increased BBB permeability. In this study, the results of our immunohistochemistry, quantitative real-time PCR and western blotting assays show a significant decrease in claudin-5 expression in the brains of SAP rats compared to that in the controls. These results are in accordance with previous studies that indicated that the downregulation of claudin-5 might contribute to the loss of integrity of the BBB.

Third, the upregulation of MMP-9 expression may contribute to BBB impairment. The expression of MMP-9 is usually upregulated in inflammatory conditions or in ischemia–hypoxia situations. It has been reported that MMP-9 induces the structural disruption of paracellular tight junctions, leading to BBB impairment in vitro [33]. Studies also showed that MMP-9 induces the degradation of collagen component [34, 35] and the downregulation of tight junction proteins [8], with increased BBB permeability under inflammatory conditions in vivo. Interestingly, our western blotting results show a noticeably increased expression level of MMP-9 in the brains of SAP rats. Thus, the upregulation of MMP-9 expression is also an important cause of the impairment of the BBB in SAP.

MSCs have been widely studied in many areas because of their pleiotropic abilities, such as multipotential differentiation, low immunogenicity and paracrine function. It has been reported that MSCs can migrate to damaged tissue and secrete trophic factors [36, 37], including cytokines and growth factors, or differentiate into functional local cells to promote tissue repair. MSCs have also been investigated for immunomodulatory abilities resulting from their secretion of various anti-inflammatory molecules [38]. MSC transplantation has been studied in several digestive diseases, in both animal models [39] and clinical trials [40]. Many studies have investigated the therapeutic effects of MSCs on acute pancreatitis [41–43], but only a few studies have focused on brain damage and investigated the underlying mechanisms.

In this study, we found that MSCs protected the BBB, decreasing its permeability in SAP rats. We explored the underlying mechanisms of MSC protective effects on the BBB and came to the following conclusions.

First, the use of MSCs in SAP rats decreases the serum levels of amylase and TNF-α and increases the serum level of IL-10, thereby alleviating pancreatic impairment. The serum amylase and TNF-α levels decreased after MSC treatment in this study, which was in accordance with the results of previous studies [11]. Decreased amylase and TNF-α levels attenuate the severity of the systemic inflammation, pancreatic impairment and the injury or apoptosis of BMECs. IL-10 is a well-known anti-inflammatory cytokine with strong immunomodulatory and anti-apoptotic abilities.

Second, MSCs reduce BMEC apoptosis levels. TUNEL staining showed that there were fewer apoptotic BMECs in SAP rats after MSC treatment. This result might mainly be attributed to the decreased serum levels of inflammatory cytokines, including TNF-α, and the increased serum IL-10 level, which was in line with a previous study [44]. It has been reported that IL-10 has anti-apoptotic effects on endothelial cells during inflammatory situations [45]. Consequently, the increased serum IL-10 levels might contribute to the anti-apoptotic effects of MSCs. In our study, the expression of Bax decreased and the expression of Bcl-2 increased in the brains of SAP rats that were treated with MSCs, indicating that MSCs have antiapoptotic effects in SAP.

Third, MSCs increase the expression level of claudin-5 in SAP rats. The results of immunohistochemistry, quantitative real-time PCR and western blotting assays show that MSCs increases claudin-5 expression in the brains of SAP rats, and supports the maintenance of BBB integrity. This result might be mainly attributed to the reduced apoptosis of BMECs in SAP rats treated with MSCs, resulting in the increased transcription and translation of claudin-5.

Fourth, MSCs decrease the expression level of MMP-9 in SAP rats. The upregulation of MMP-9 expression is associated with increased BBB permeability, as mentioned above. The results of western blotting assays showed that the expression of MMP-9 was downregulated by MSCs in SAP rats. This result may be due to an MSC-mediated decrease in the serum levels of inflammatory cytokines because the transcription and translation of MMP-9 are often initiated by inflammatory cytokines during inflammatory situations [46].

The method of MSCs infusion, including intravenous and intraarterial infusion might exert different effects on the BBB in SAP rats. Intraarterial infusion of MSCs increases the local presence of MSCs in cerebral circulation, but is more difficult than intravenous infusion and has higher risks of microvascular embolization. However, MSCs cannot migrate through the BBB due to their large size and the barrier properties. Therefore, the protective effects of MSCs on the BBB in SAP rats might be mainly attributed to the immunomodulatory and paracrine functions of MSCs. Notably, MSC-derived exosomes are also currently a research focus in cell-free regenerative medicine due to them having similar biological effects but without the ethical issues of cell transplantation. Additional research that further explores the underlying molecular mechanisms will be required in the future.

## Conclusions

We found that in SAP rats, increased levels of amylase and inflammatory cytokines, BMEC apoptosis, downregulation of claudin-5 and upregulation of MMP-9 might be the main mechanisms driving the impairment of the BBB, including increased permeability. However, MSCs attenuated the severity of systematic inflammation and pancreatic impairment, reduced BMEC apoptosis, upregulated claudin-5 and downregulated MMP-9, decreasing BBB permeability in SAP rats.

## Additional file

Additional file 1: Table of primers. (DOCX 15 kb)

## Data Availability

The data sets supporting the results of this article are included within the article.

## Abbreviations

Bax: Bcl-2-associated X
BBB: Blood–brain barrier
Bcl-2: B-cell lymphoma 2
BMEC: Brain microvascular endothelial cell
CLS: Capillary leak syndrome
DMEM: Dulbecco’s modified Eagle’s medium
ELISA: Enzyme-linked immunosorbent assay
FBS: Fetal bovine serum
GAPDH: Glyceraldehyde-3-phosphate dehydrogenase
H&E: Hematoxylin and eosin
IL-10: Interleukin-10
MMP-9: Matrix metalloproteinase-9
MSC: Mesenchymal stem cell
PBS: Phosphate-buffered saline
PE: Pancreatic encephalopathy
SAP: Severe acute pancreatitis
SD: Sprague-Dawley
SIRS: Systemic inflammatory response syndrome
TNF-α: Tumor necrosis factor-α
TUNEL: Terminal-deoxynucleotidyl transferase-mediated nick end labelling
