# Correction to: Mesenchymal stem cells decrease blood–brain barrier permeability in rats with severe acute pancreatitis

**DOI:** 10.1186/s11658-019-0176-7

**Published:** 2019-08-26

**Authors:** Ronggui Lin, Ming Li, Meiqin Luo, Tianhong Teng, Yu Pan, Heguang Huang

**Affiliations:** 1 Department of General surgery, Fujian Medical University Union Hospital, 29 Xinquan Road, Fuzhou, Fujian 350001 People’s Republic of China; 2 Department of Histology and Embryology, Hunan University of Medicine, Huaihua, Hunan China; 3 Department of Orthopedics, Fujian Medical University Union Hospital, Fuzhou, Fujian China

**Correction to: Cell Mol Biol Lett (2019) 24:43**

**https://doi.org/10.1186/s11658-019-0167-8**

Following publication of the original article [1], the author informed us that Fig. 5 was incorrect.

**Fig. 5:**
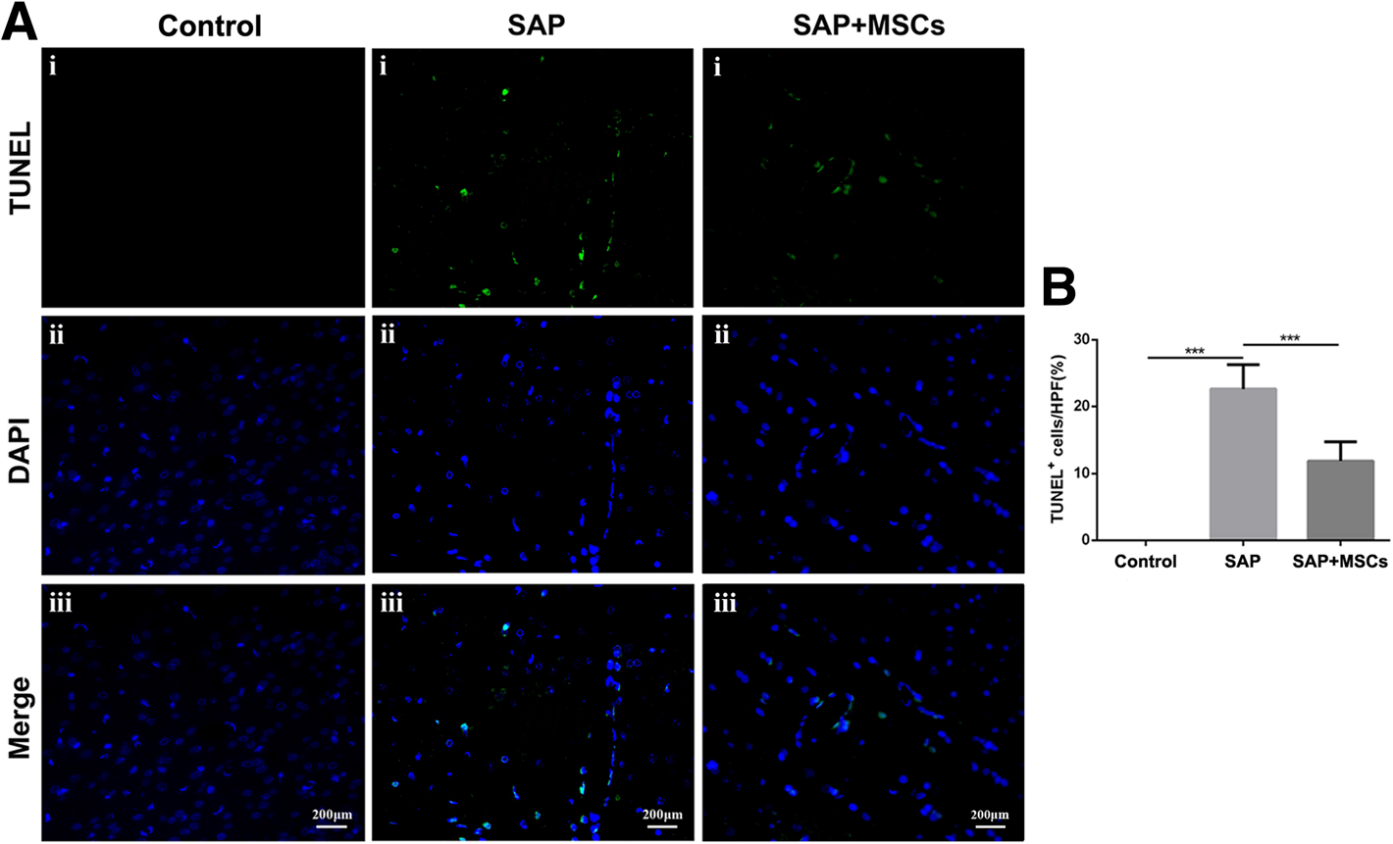
MSCs reduced BMEC apoptosis in the brains of SAP rats**. a** TUNEL staining of the brain, scale bar = 200 μm. No apoptosis was observed in the control group, but numerous apoptotic cells, mainly BMECs, were observed in the SAP group. Fewer apoptotic cells were observed in the SAP+MSCs group than in the SAP group. **b** Statistical analysis of TUNEL-positive cells. ***p < 0.001

The correct figure is given below.
