# Supplementary material for: Mesenchymal stem cells decrease blood–brain barrier permeability in rats with severe acute pancreatitis
Source: Cell Mol Biol Lett. 2019 Jun 17;24:43. doi: 10.1186/s11658-019-0167-8 (PMC6580617; doi:10.1186/s11658-019-0167-8)
Supplement: Supplementary file 1 — Table of primers. (DOCX 15 kb) [file 11658_2019_167_MOESM1_ESM.docx]

| GAPDH | NM_017008.4 Rattus norvegicus glyceraldehyde-3-phosphate dehydrogenase (Gapdh), mRNA  Forward primer 1 GCGAGATCCCGCTAACATCA 20  Template 306 ......................................... 325  Reverse primer 1 CTCGTGGTTCACACCCATCA 20  Template 483 ........................................ 464 |
| --- | --- |
| Claudin 5 | NM_031701.2 Rattus norvegicus claudin 5 (Cldn5), mRNA  Forward primer 1 GCACTCTTTGTTACCTTGAC 20  Template 422 …………………………………….. 441  Reverse primer 1 GGCACCGTTGGATCATAG 18  Template 600 …………………………….…... 583 |
| Bax | NM_017059.2 Rattus norvegicus BCL2 associated X, apoptosis regulator (Bax), mRNA  Forward primer 1 CAGACGGCAACTTCAACT 18  Template 391 …………………………………….. 408  Reverse primer 1 CTTCCAGATGGTGAGTGA 18  Template 656 …………………………….…….. 639 |
| Bcl-2 | NM_016993.1 Rattus norvegicus BCL2, apoptosis regulator (Bcl2), mRNA  Forward primer 1 GCAGAGATGTCCAGTCAG 18  Template 562 …………………………………….. 579  Reverse primer 1 ATCCACAGAGCGATGTTG 18  Template 755 …………………………….…….. 738 |
